# Plasma Membrane Ca^2+^ Pump PMCA4z Is More Active Than Splicing Variant PMCA4x

**DOI:** 10.3389/fncel.2021.668371

**Published:** 2021-08-26

**Authors:** Gerardo R. Corradi, Luciana R. Mazzitelli, Guido D. Petrovich, Felicitas de Tezanos Pinto, Lucia Rochi, Hugo P. Adamo

**Affiliations:** Departamento de Química Biológica, Instituto de Química y Fisicoquímica Biológicas, Facultad de Farmacia y Bioquímica, Consejo Nacional de Investigaciones Científicas y Técnicas-Universidad de Buenos Aires, Buenos Aires, Argentina

**Keywords:** calcium transport, calcium ATPase, PMCA isoforms, neuronal PMCA, heart PMCA

## Abstract

The plasma membrane Ca^2+^ pumps (PMCA) are P-ATPases that control Ca^2+^ signaling and homeostasis by transporting Ca^2+^ out of the eukaryotic cell. Humans have four genes that code for PMCA isoforms (PMCA1-4). A large diversity of PMCA isoforms is generated by alternative mRNA splicing at sites A and C. The different PMCA isoforms are expressed in a cell-type and developmental-specific manner and exhibit differential sensitivity to a great number of regulatory mechanisms. PMCA4 has two A splice variants, the forms “x” and “z”. While PMCA4x is ubiquitously expressed and relatively well-studied, PMCA4z is less characterized and its expression is restricted to some tissues such as the brain and heart muscle. PMCA4z lacks a stretch of 12 amino acids in the so-called A-M3 linker, a conformation-sensitive region of the molecule connecting the actuator domain (A) with the third transmembrane segment (M3). We expressed in yeast PMCA4 variants “x” and “z”, maintaining constant the most frequent splice variant “b” at the C-terminal end, and obtained purified preparations of both proteins. In the basal autoinhibited state, PMCA4zb showed a higher ATPase activity and a higher apparent Ca^2+^ affinity than PMCA4xb. Both isoforms were stimulated by calmodulin but PMCA4zb was more strongly activated by acidic lipids than PMCA4xb. The results indicate that a PMCA4 intrinsically more active and more responsive to acidic lipids is produced by the variant “z” of the splicing site A.

## Introduction

The Ca^2+^ transporting P-ATPases known as Ca^2+^ pumps are key elements for the control of intracellular Ca^2+^ homeostasis (Chen et al., 2020). Animal cells possess three types of Ca^2+^ pumps, the endoplasmic-sarcoplasmic reticulum (SERCA), the secretory pathway (SPCA), and the plasma membrane Ca^2+^ pump (PMCA). By taking Ca^2+^ out of the cell, the PMCA modulates the resting cytosolic Ca^2+^ and is fundamental for shaping and ending the Ca^2+^ signals (Lopreiato et al., 2014; Stafford et al., 2017). The relative contribution of the PMCA to these processes depends on the cell type and the activity of other Ca^2+^ transporters. In excitable cells, the SERCA pump and the plasma membrane Na^+^/Ca^2+^ exchanger (NCX) are mostly responsible for removing Ca^2+^ from the cytosol, while the function of PMCA seems more suited for the control of Ca^2+^ dependent microdomain processes (Strehler, 2015). Consistent with this idea, in excitable cells, PMCA is a part of protein networks that by including voltage-gated Ca^2+^ channels, couple influx, and clearance of Ca^2+^ (Müller et al., 2010). The loss of PMCA function has been associated with several human diseases, including neurologic pathologies, such as familial spastic paraplegia (Mohamed et al., 2011, 2016; Li et al., 2014).

In humans four genes code for PMCA proteins (PMCA1-4) with a calculated molecular mass of 135 kDa and an average sequence identity of about 80% (Lopreiato et al., 2014). The expression profile of the PMCA isoforms varies depending on the tissue and the stage of development. PMCA1 and PMCA4 have ubiquitously expressed forms while the expression of PMCA2 and PMCA3 is more restricted and particularly abundant in nervous tissues (Strehler and Zacharias, 2001). While neurons are rich in all PMCA isoforms and variants, PMCA4 constitutes a major one at the early stages of the differentiation process and is markedly enriched, together with PMCA1, and PMCA2 at the postsynaptic membrane through the interaction with the postsynaptic density protein 95 (Kim et al., 1998; Boczek et al., 2019).

**Figure 1:**
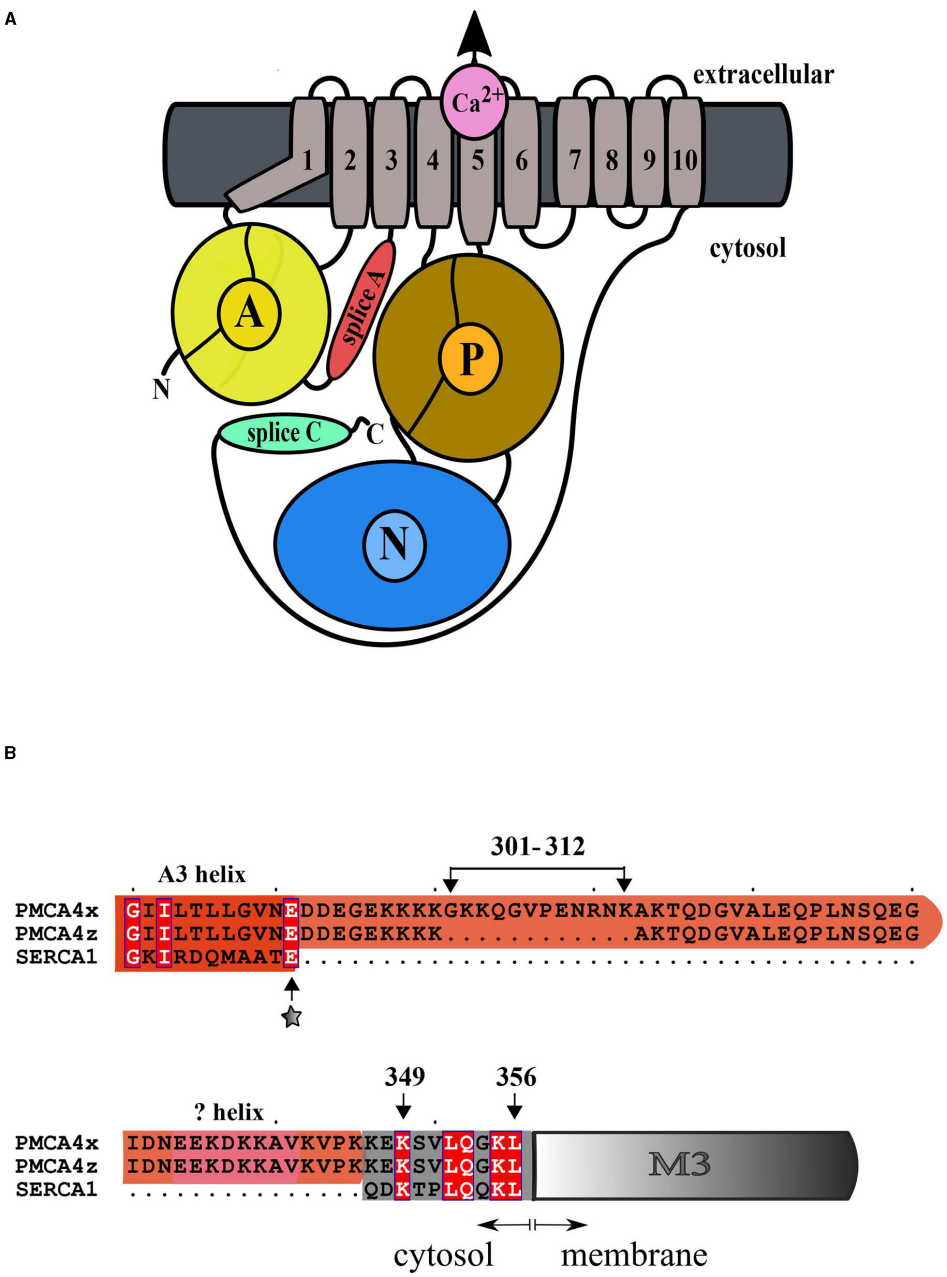
**(A)** General architecture of the plasma membrane Ca^2+^ (PMCA) P-ATPase. Only the catalytic subunit is represented. Helices M1-M6 constitute the transport domain. The action domain (A) is shown in yellow, the phosphorylation domain (P) is brown and the nucleotide-binding domain (N) is light blue. The A-M3 linker, which is modified by mRNA splicing at site A, is shown in red and the regulatory C-terminal domain, which is modified by mRNA splicing at site C, is shown in green. **(B)** Alignment of the amino acid sequence of the A-M3 linker of PMCA4x, PMCA4z, and SERCA1. Conserved residues are in white bold. The “x” insertion of amino acids 301–312 is shown. The position of the A3 (red) and A_L_ helices which are predicted by modeling (pink) are indicated. The transmembrane M3 helix is colored gray and its membrane inserted portion is shown as a box. The site of SERCA mutational insertion (site 1) described by Nyholm Holdensen and Andersen (2009) is indicated by a star.

Recently, the Cryo-EM structure of the human PMCA1 in complex with neuroplastin was reported (Gong et al., 2018). As expected, the general architecture of the PMCA protein closely resembles that of other P-ATPases with the characteristic N, nucleotide binding, P phosphorylation, and A, action domain (Figure 1A). PMCA forms heteromers with the glycoproteins neuroplastin or basigin in different cell types (Korthalsa et al., 2017; Schmidt et al., 2017). Contacts between PMCA and neuroplastin were observed at the PMCA extracellular M8-M9 linker and with the M10 transmembrane segment. The interaction of the PMCA with neuroplastin takes place in the ER and affects PMCA stability, function, and plasma membrane targeting. These findings open the interesting possibility that the activity of the PMCA can influence neuroplastin functions, for example, synaptic plasticity and neuritogenesis (Beesley et al., 2014).

A distinctive characteristic of the PMCA family of Ca^2+^ pumps is the autoinhibitory mechanism and the direct stimulation by calmodulin (CaM) (Hegedus et al., 2020). Autoinhibition is mediated by the interaction of the regulatory domain, located in the extended C-terminal tail with the central catalytic core of the protein. The binding of Ca^2+^-CaM to the C-terminal regulatory domain releases the PMCA from the autoinhibition (Adamo and Grimaldi, 1998; Bredeston and Adamo, 2004; Corradi and Adamo, 2007; Mazzitelli and Adamo, 2014; Hegedus et al., 2020).

A second important mechanism of PMCA regulation is the Ca^2+^-independent activation by acidic lipids (Niggli et al., 1981; Enyedi et al., 1987; Brodin et al., 1992; Filoteo et al., 1992; de Tezanos Pinto and Adamo, 2002, 2006). Although this mechanism has not been explained, part of the stimulatory effect of acidic lipids is due to their interaction with the C-terminal regulatory domain (Brodin et al., 1992; Filoteo et al., 1992). However, because some acidic lipids, in particular phosphoinositides, were found more effective than calmodulin to activate the PMCA at low concentrations of Ca^2+^ (Enyedi et al., 1987) a second site was proposed, the so-called acidic lipid (A_L_) responsive region, in the segment that links domain A with the transmembrane helix M3 (Brodin et al., 1992; Filoteo et al., 1992; de Tezanos Pinto and Adamo, 2002, 2006).

Besides the four PMCA genes, many PMCA variants are generated by the combination of the products of alternative splicing of mRNAs at the two sites called sites A and C. While these splice variations do not affect the basic Ca^2+^ transport function, they are strategically located in the regulatory regions of the PMCA molecule and hence they may affect the maximal transport capacity and Ca^2+^ affinity as well as the interaction of the PMCA with the other regulatory proteins (Strehler and Zacharias, 2001; Lopreiato et al., 2014). Splicing at site C changes the C-terminal regulatory tail, generating the most common variants “a” and “b” and others identified as “c” through “g”, some of which have not yet been confirmed at the protein level. The degree of autoinhibition and the kinetics of CaM activation are unique features of each PMCA isoform and are modified by the alternative C-terminal sequences of each isoform variant (Enyedi et al., 1994; Strehler, 2015).

In humans, splicing at site A results in three PMCA variants, the forms “z”, “x”, and “w”, which have insertions of 0, 12, and 45 extra amino acids in the A_L_ region, respectively. PMCA1 contains only the “x” variant, while in PMCA2-4 variants “x” and “z” have been detected. In humans, only PMCA2 has the longest “w” insertion (Strehler and Zacharias, 2001; Hegedus et al., 2020) while in the rat in addition to “w”, an “y” variant with the insertion of the first 31 residues has been detected (Adamo and Penniston, 1992). Because these insertions modify the A_L_ region, previous studies have suggested that different splice A variants may differ in their sensitivity to acidic lipid activation (Brodin et al., 1992; Filoteo et al., 1992; de Tezanos Pinto and Adamo, 2002, 2006).

At present, only few PMCA forms have been individually characterized and the functional consequences of some splice variants are still controversial. Furthermore, the effect of the splicing variants may depend on the context of the particular PMCA isoform considered. In this study, we have characterized the enzymatic activity of purified preparations of the site A splice variants of PMCA4 which comprise two forms (Figure 1B), the ubiquitously expressed form “x” and the shorter variant “z” showing a more restricted expression profile, particularly, in the brain and heart. We found that PMCA4z is more active and more responsive to activation by acidic lipids than the PMCA4x.

## Materials and Methods

### Chemicals

Yeast synthetic drop-out media supplement without leucine, yeast nitrogen base without amino acids, dextrose, polyoxyethylene 10 lauryl ether (C_12_E_10_), L-α-phosphatidylcholine type XVI-E (Sigma) from fresh egg yolk, brain extract (BE) Type I Folch Fraction I from bovine brain containing approximately (w/w) 10% PI, 50% PS, and other lipids, phosphodiesterase 3′, 5′ cyclic nucleotide activator (calmodulin) from bovine brain, calmodulin-agarose, ATP (disodium salt, vanadium-free), sodium dodecyl sulfate (SDS), and all other chemicals were obtained from Sigma.

### Construction of DNA Coding for PMCA4zb

The cDNA coding for PMCA4zb was obtained by a two-step PCR using Pfu DNA polymerase and the PMCA4xb cDNA as template. During the first step, two PCR reactions were run, one using primer His: 5′CTGGAGGTCGACATGACGCATCACCATCACCATCACAACCCATCAGACCGTGTCTTGCCTGCCAA3′, and N-term 5′TTTCTTCTTTTTCTCCCCTTCGTCATC3′, and the other using primer 2096: 5′TGACAACATCAACACAGCCCGGGCCATTGCCACCA3′ and C-term 5′GATGACGAAGGGGAGAAAAAGAAAGCAAAGACCCAAGACGGAGTG3′. The primer His contains a restriction site for nuclease SalI that matches a unique site for SalI at the 5′position of the PMCA4xb cDNA, while primer 2096 anneals to the PMCA4xb DNA downstream of a naturally occurring BspEI unique site. The 930-bp His-N-term and 1210-bp C-term-2096 products were combined in the next PCR step along with primers His and 2096. The amplified 2119-bp His-2096 fragment was digested with SalI and BspEI and used to replace the corresponding sequence of PMCA4xb GFP DNA cloned into pMP625 (Bredeston and Adamo, 2004) or pYX112 (Mazzitelli and Adamo, 2014) expression vectors.

### Complementation Assay on K616 *Saccharomyces cerevisiae* Strain

K616 cells (MATα pmr1::HIS3 pmc::TRP1 cnb1::LEU2, ura3) (Cunningham and Fink, 1994) were transformed using a lithium acetate/polyethylene glycol method (Elble, 1992) with expression vector pYX112 containing different PMCA constructs, and the transformants were selected for their ability to grow in the absence of uracil on plates containing 6.7% YNB, 0.67% complete supplemented medium minus Ura (Ura-), 2.2% dextrose, 0.05% 2N-morpholino-ethanesulfonic acid (MES) pH = 6.0, 10 mM Cl_2_Ca, and 1.5% agar. For complementation studies, 15 μl of log-phase was streaked onto plates lacking uracil and containing either 10 mM Cl_2_Ca or 10 mM ethylene glycol-bis-N,N,N′,N′-tetra acetic acid (EGTA). The plates were allowed to grow for 72 h at 28°C.

### Plasma Membrane Ca^2+^ Pump Expression and Purification

*Saccharomyces cerevisiae* strain DBY2062 (MATa his4-619 leu2-3,112) (Bauer and Kolling, 1996) was used for expression and purification. Yeast cells were transformed with vector pMP625 containing a Leu^+^ marker and the PMA1 promoter. Yeast cells were transformed and selected in the absence of leucine. PMCA4xb-GFP and PMCA4zb-GFP were purified as described previously (Bredeston and Adamo, 2004; Mazzitelli and Adamo, 2014). Briefly, 4 L of yeast (A600 ~ 4.0–5.0) were solubilized with C_12_E_10_ and the solubilizate was loaded onto a calmodulin-agarose chromatography column. After washing with a purification buffer containing Ca^2+^, the PMCA protein was eluted with EGTA. Lipids were not added during the purification.

### Calcium-ATPase Activity

The Ca^2+^-ATPase activity of the purified preparations of PMCA4xb-GFP and PMCA4zb-GFP was estimated from the release of [^32^P]Pi from [γ-^32^P]ATP at 28°C as described previously (Mazzitelli and Adamo, 2014). The reaction medium contained 20 mM HEPES-K (pH = 7.2 at 28°C), 100 mM KCl, 4 mM MgCl_2_, 3 mM ATP, 500 μM EGTA, and enough CaCl_2_ to give the Ca^2+^ concentrations indicated in each experiment. The concentration of free Ca^2+^ in the reaction medium was calculated using the WebmaxC Standard program. Before starting the ATPase reaction, 1 μg of the enzyme (50 μl) was gently stirred for 5 min with 5 μl of a sonicated mixture of 57 mg/ml C_12_E_10_ with 29 mg/ml PC or the indicated amount of BE. The suspension was thoroughly mixed and preincubated for at least 10 min on ice before being added to the ATPase reaction. The PMCA was incubated for 3 min at 37°C with the reaction medium before the initiation of the reaction by adding 3 mM [γ-32P]ATP. The reaction was finished by acid denaturation after 30 min. During this period, the amount of Pi released from ATP increased linearly with time.

### Sodium Dodecyl Sulfate–Polyacrylamide Gel Electrophoresis, Protein Quantitation and Western Blotting

Proteins were electrophoresed on a 7.5% acrylamide gel according to Laemmli (Laemmli, 1970) and revealed by Coomassie Blue staining as previously described (Mazzitelli and Adamo, 2014). The protein concentration was initially estimated by the method of Bradford (Bradford, 1976) using bovine serum albumin as standard. The content of PMCA protein in each preparation was estimated by SDS–PAGE comparing the intensity of the bands of PMCA protein with different amounts of bovine serum albumin as standard, stained with Coomassie Blue.

Western blotting was performed as previously described (Cura et al., 2008). Briefly, after SDS–PAGE the proteins were electrotransferred onto Millipore Immobilon P membranes and the non-specific binding was blocked by incubating the membranes overnight at 4°C in a solution of 160 mM NaCl, 0.05% Tween 20, and 1% non-fat dry milk. The membranes were incubated for 1 h with anti-PMCA monoclonal antibodies (Adamo et al., 1992; Caride et al., 1996) from ascitic fluid (dilution 1:1,000) and developed using biotinylated antimouse immunoglobulin G and avidin-horseradish peroxidase conjugate.

### Data Analysis

Data presented in this work are representative of at least three independent experiments performed in duplicate. The kinetic data were analyzed by non-linear curve fitting using the SigmaPlot 10 scientific data analysis and graphing software (Systat Software Inc.). The same model based on the Hill equation was fitted to PMCA4xb and PMCA4zb and the statistical significance of the difference between datasets was assessed by the *F*-test as determined by the OriginLab2019 software.

## Results

### The Isoform h4z Complements the Mutant Yeast K616

**Figure 2:**
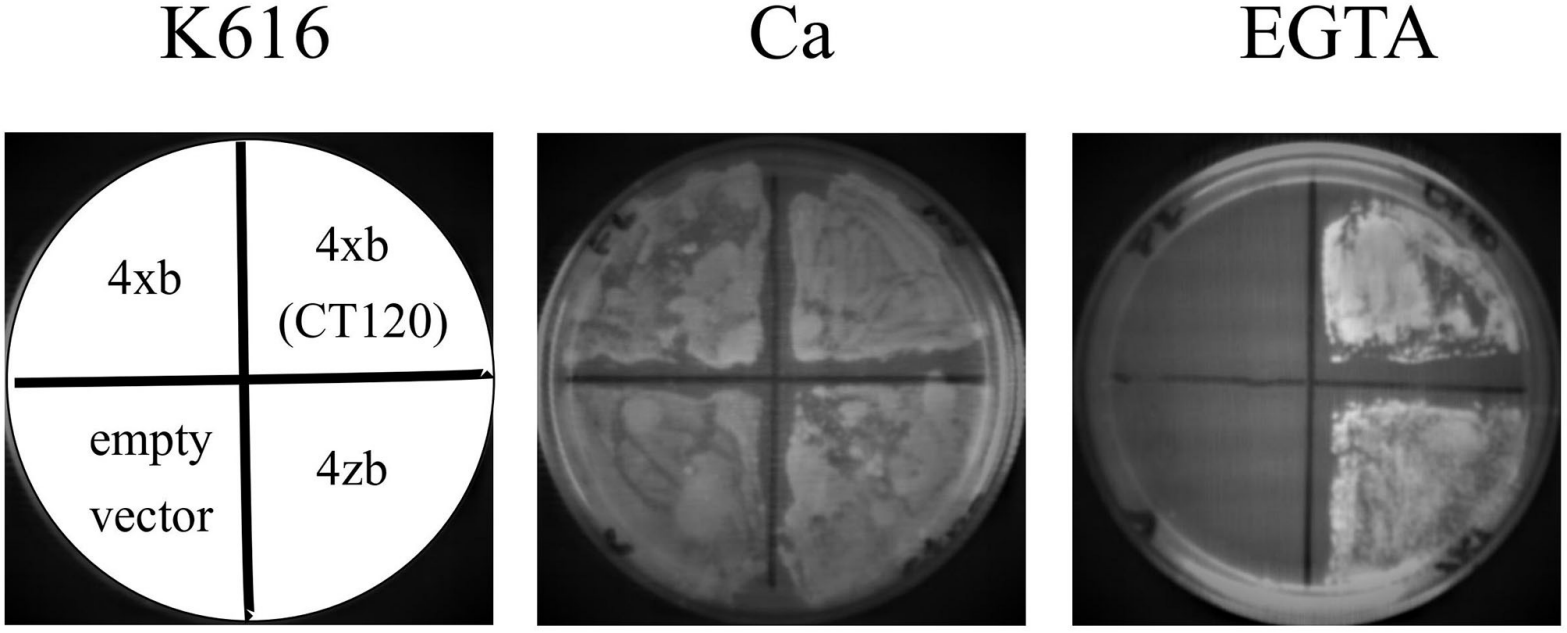
PMCA4xb-GFP complements the K616 yeast strain lacking endogenous Ca^2+^ ATPases. K616 cells were transformed with the empty pYX112 vector, or vector containing PMCA4xb-GFP, PMCA4zb-GFP, or the hyperactive mutant PMCA4x(CT120)-GFP lacking the C-terminal 120 amino acids segment containing the autoinhibitory domain. Cultures of K616 yeast expressing the recombinant PMCA proteins were grown for 72 h at 28°C on SC-Ura^−^ plates containing 10 mM CaCl_2_ or 10 mM EGTA.

In the *S. cerevisiae* strain K616, the genes coding for Ca^2+^ ATPases PMR1, PMC1, and the regulatory subunit of calcineurin (CNB) have been inactivated (Cunningham and Fink, 1994). K616 cells cannot proliferate in media containing low concentrations of Ca^2+^ (10 mm EGTA), but this phenotype can be rescued by the expression of a high-affinity Ca^2+^ transport system. We have previously shown that the expression of PMCA4xb does not allow the growth of K616 cells in Ca^2+^ depleted media and successful complementation requires the expression of hyperactive forms of the protein, in which the autoinhibitory mechanism has been disrupted Activated versions of PMCA4xb can be produced either by introducing mutations in the A domain or by the removal of the C-terminal autoinhibitory segment, as in the case of the mutant CT120 lacking the C-terminal 120 amino acid residues (Enyedi et al., 1993; Bredeston and Adamo, 2004; Mazzitelli and Adamo, 2014). As shown in Figure 2, K616 cells transformed with the empty vector, and a vector containing DNA coding for PMCA4xb, PMCA4xCT120, and PMCA4zb, successfully grew in selective media containing 10 mM CaCl_2_. However, only cells transformed with PMCA4xCT120 and PMCA4zb were able to grow in the presence of 10 mM EGTA. This result suggested that, such as the PMCA4xCT120 mutant, the PMCA4zb is hyperactive and has a higher Ca^2+^ pumping activity than PMCA4xb.

### Subcellular Localization of the Human PMCA4xb and PMCAzb Expressed in Yeast Cells

**Figure 3:**
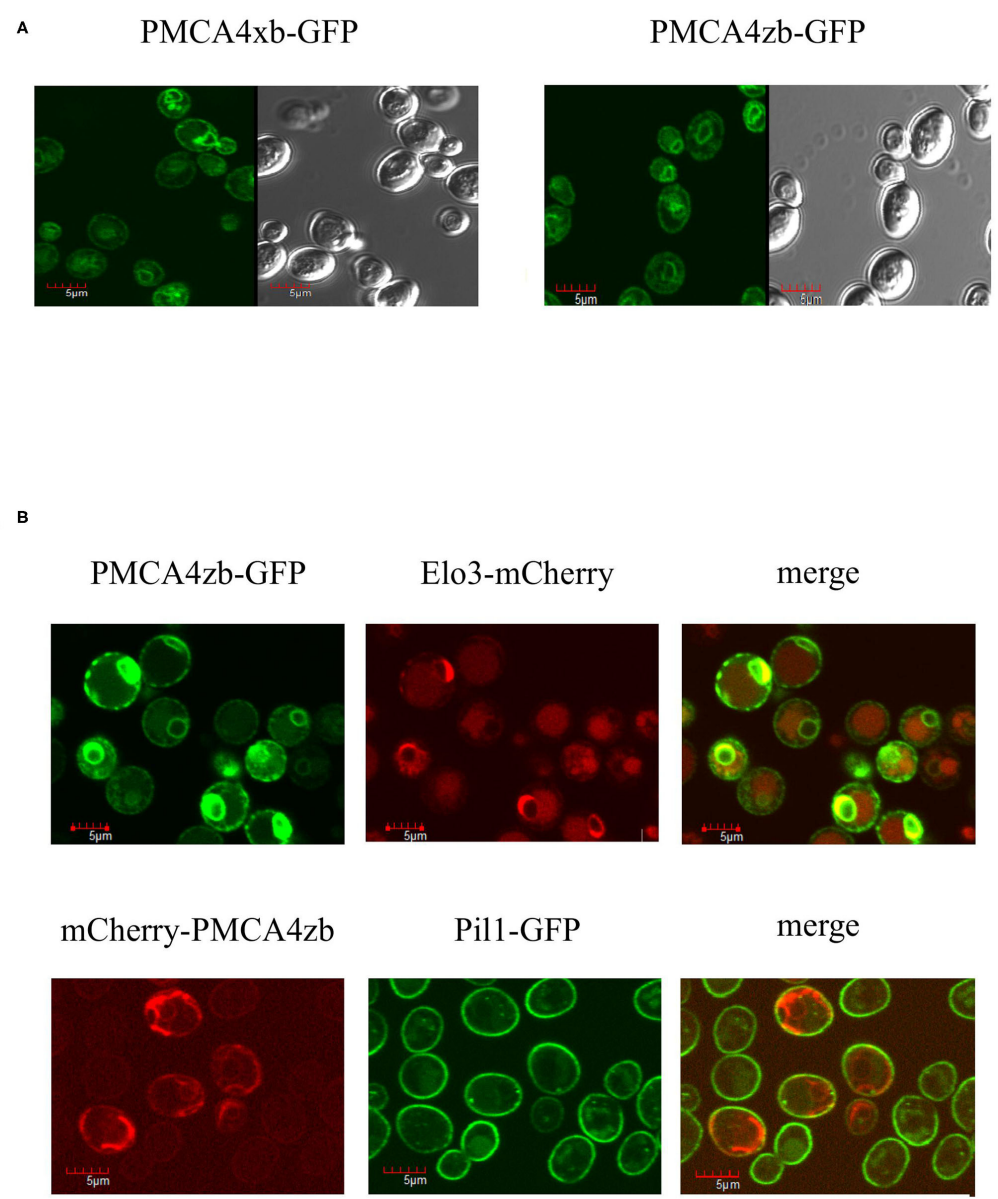
**(A)** Expression of PMCA4xb and PMCA4zb-GFP in yeast cells. *Saccharomyces cerevisiae* cells (strain DBY2062) were transformed with the pMP625 vector containing the DNA coding for PMCA4xb-GFP and PMCA4zb-GFP and selected in a Leu^−^ media. The confocal fluorescent image (EGFP filter, LUT 2300, amplification x60) is shown on the left. The prominent yeast vacuoles can be distinguished in the visible image shown on the right. **(B)** Intracellular localization of the expressed PMCA4zb. *On top*, PMCA4zb-GFP was coexpressed with the ER membrane protein Elo3-mCherry. The observed distribution suggests that both proteins localize in the perinuclear and peripheral ER membranes. Some background mCherry fluorescence is also detected in vacuoles. *On the bottom*, mCherry-PMCA4zb was coexpressed with the plasma membrane protein Pil1-GFP. The mCherry fluorescence suggests that most of the PMCA protein in the cell periphery does not reach the plasma membrane.

Using the GFP fusions, we determined the localization of the expressed PMCA4 proteins by confocal microscopy. As shown in Figure 3A, both PMCA4xb and PMCA4zb exhibited a similar pattern of fluorescence intensity in the cell periphery and around the nucleus, suggesting that they localized in the membranes of the endoplasmic reticulum. Confirming this idea, PMCA fluorescence showed significant colocalization with the endoplasmic reticulum protein-membrane ELO3 and very little with PIL1 at the plasma membrane (Figure 3B).

### Purified Preparations of PMCA4xb and PMCA4zb

**Figure 4:**
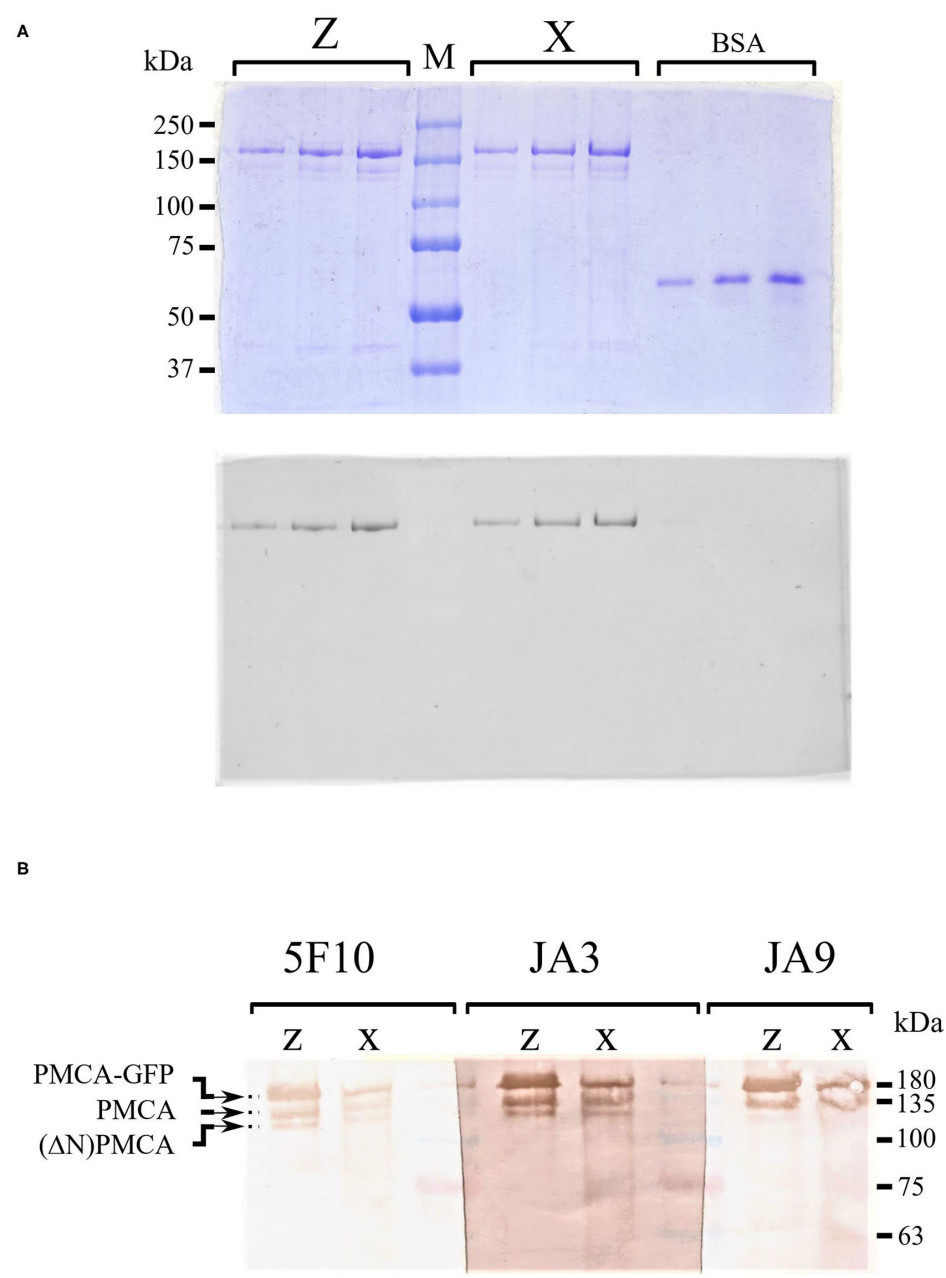
**(A)** Sodium dodecyl sulfate–polyacrylamide gel electrophoresis (SDS-PAGE) of purified PMCA4xb-GFP and PMCA4zb-GFP. The recombinant PMCA proteins were purified as described in “Materials and methods.” Aliquots of the eluate containing the purified proteins were submitted to electrophoresis in a 7.5% SDS–PAGE. Three lanes containing 0.25, 0.5, and 0.75 μg of PMCA4xb-GFP and PMCA4zb-GFP are shown. For comparison 0.3, 0.6, and 0.9 μg of bovine serum albumin were run on the right side of the gel. *On top*, the gel was stained with Coomassie-blue. *On the bottom*, the GFP-fluorescence of the same gel is shown. **(B)** Western blots of purified PMCA4xb-GFP and PMCA4zb-GFP. Western blots of purified PMCA4 preparations with antibodies 5F10, JA3, and JA9. Aliquots of 6 μl (about 1 μg of protein) of PMCA4xb-GFP and PMCA4zb-GFP eluates were loaded.

The PMCA proteins were purified from *S. cerevisiae* membranes by calmodulin affinity chromatography as described previously (Bredeston and Adamo, 2004; Corradi and Adamo, 2007; Cura et al., 2008; Mazzitelli and Adamo, 2014). Analysis by SDS–PAGE showed that both purified preparations contained a major peptide of an estimated Mr 180 kDa corresponding to the PMCA-GFP fusions and small amounts of two faster-migrating fragments of about 140 and 125 kDa. These shorter fragments were likely the result of the PMCA-GFP proteolysis at the C-terminal region because they lacked GFP fluorescence (Figure 4A). Both PMCA4xb-GFP and PMCAzb-GFP showed similar proteolytic peptides. We further analyzed the purified preparations by Western blot using anti-PMCA specific antibodies 5F10, JA3, and JA9 (Adamo et al., 1992; Caride et al., 1996). The 5F10 epitope is between PMCA4xb residues 719–738 in the central cytosolic loop of the molecule, while JA3 and JA9 recognize the C and N-terminal segments at residues 1156–1180 and 51–75, respectively. Figure 4B shows that antibodies 5F10 and JA3 detected three PMCA peptides that, based on their migration, corresponded to the full-length PMCA-GFP and two shorter proteolytic products. JA9 recognized the PMCA-GFP full-length peptide and only the larger proteolytic fragment. This proteolysis pattern suggests that the large proteolytic fragment of 140 kDa that was recognized by the three antibodies corresponded to the PMCA proteins and was produced by the removal of GFP from the PMCA-GFP. The proteolytic peptide of 125 kDa, would be produced by the cleavage of a segment from the N-terminal region of PMCA because it was no longer detected by JA9.

### Adenosine Triphosphatase Activity and Ca^2+^ Dependency of PMCA4xb and PMCA4zb

**Figure 5:**
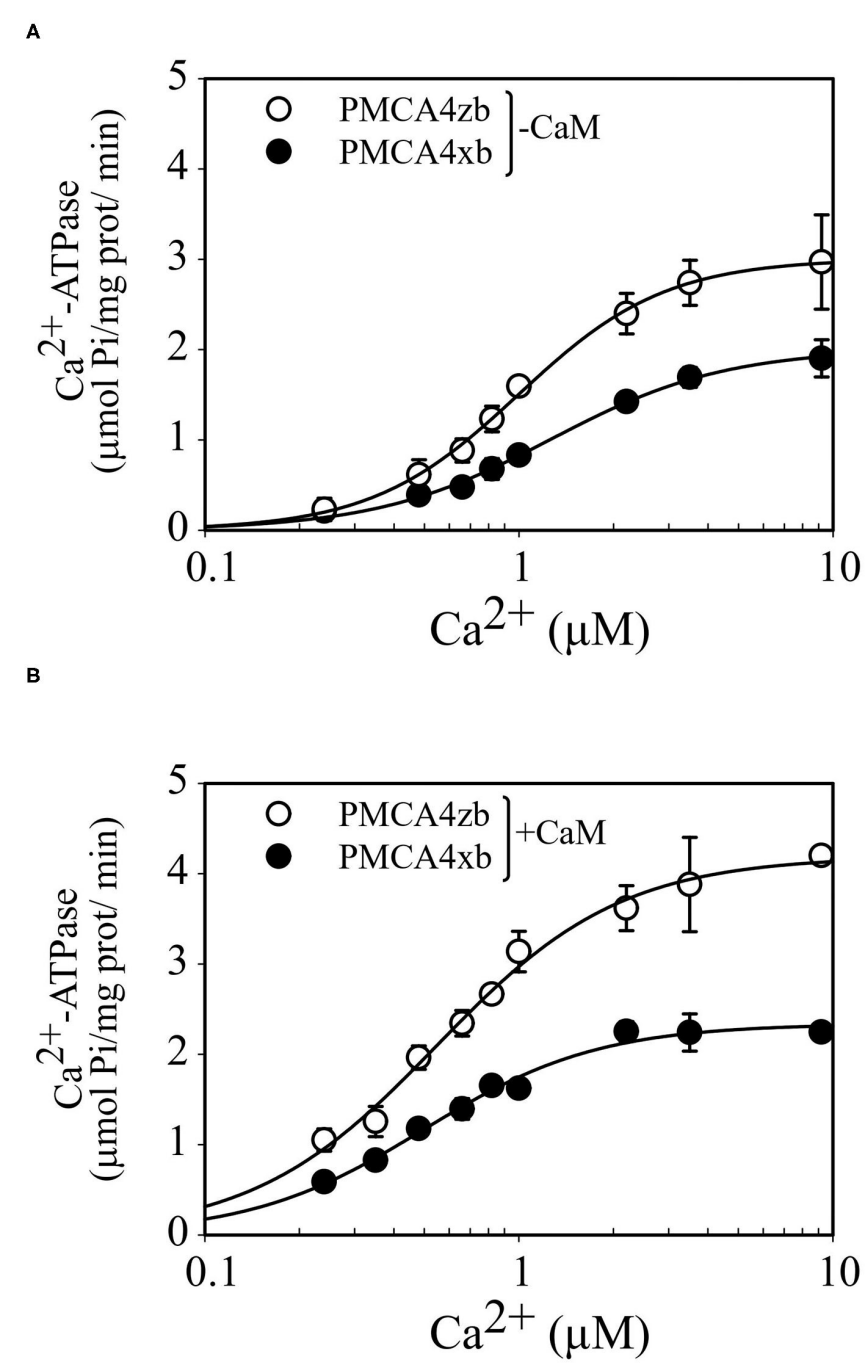
Ca^2+^ dependence of the Ca^2+^-ATPase activity of purified PMCA4zb-GFP and PMCA4xb-GFP in the absence **(A)** and the presence of calmodulin **(B)**. The ATPase reaction was measured at 28°C as described previously (Mazzitelli and Adamo, 2014). The reaction media contained 1 μg of PMCA supplemented with PC, 50 mM HEPES-K (pH = 7.2 at 28°C), 100 mM KCl, 4 mM MgCl_2_, 3 mM ATP, 500 μM EGTA, and enough CaCl_2_ to give the indicated concentrations of Ca^2+^. When calmodulin was present its final concentration was 200 nM. The reaction time was 15 min. The data points are from a representative experiment conducted in duplicate. *Error bars* show the SD. The lines are the best fit to the data given by the Hill equation *v* = Vmax *[Ca]^nH^/([K_0.5Ca_]^nH^ + [Ca]^nH^) using the values of the parameters shown in Table 1.

The purified and partially delipidated PMCA4xb and PMCAzb were reactivated by the addition of phosphatidylcholine before the Ca^2+^-ATPase activity was measured at increasing concentrations of Ca^2+^. As shown in Figure 5, the activity of PMCA4zb was higher than that of PMCA4xb in all the range of Ca^2+^ concentrations tested. Kinetic analysis indicated that the higher activity of PMCA4zb was a consequence of the increase of the Vmax and the apparent affinity for Ca^2+^ (Table 1). The addition to the reaction media of a high saturating amount of CaM increased the Vmax and decreased the Ca^2+^ apparent affinity of PMCA4xb and PMCA4zb. Both, in basal conditions and the CaM-activated state, PMCA4zb exhibited 1.5–2-fold higher maximal activity than PMCA4xb. However, in the CaM-activated state, the apparent affinity for Ca^2+^ of both variants was similar.

**Table 1:** Comparison of the Ca^2+^ dependency of purified PMCA4 isoforms.

	**K_**0.5Ca**_ (μM)**	**Vmáx** **(μmol/mg/min)**	**nH**	**Catalytic efficiency (μM.s^**−1**^)**
PMCA4xb-GFP	1.26 ± 0.08	2.01 ± 0.06	1.6 ± 0.1	35
PMCA4zb-GFP	0.99 ± 0.03	3.00 ± 0.06	1.8 ± 0.1	68
PMCA4xb-GFP+CaM	0.50 ± 0.01	2.33 ± 0.07	1.6 ± 0.2	105
PMCA4zb-GFP+CaM	0.55 ± 0.04	4.19 ± 0.12	1.5 ± 0.2	172

*The kinetic constants were estimated by the non-linear fitting of the Hill equation to the data of Ca^2+^-ATPase activities shown in Figure 4. The values of the parameters ± SE as given by the fitting program are shown. The catalytic efficiency was estimated by the ratio Vmax/K_0.5Ca_. According to the F-test, at the 0.05 significance level, the datasets of PMCA4xb and PMCA4zb are statistically different*.

### Dependence With Acidic Lipids

**Figure 6:**
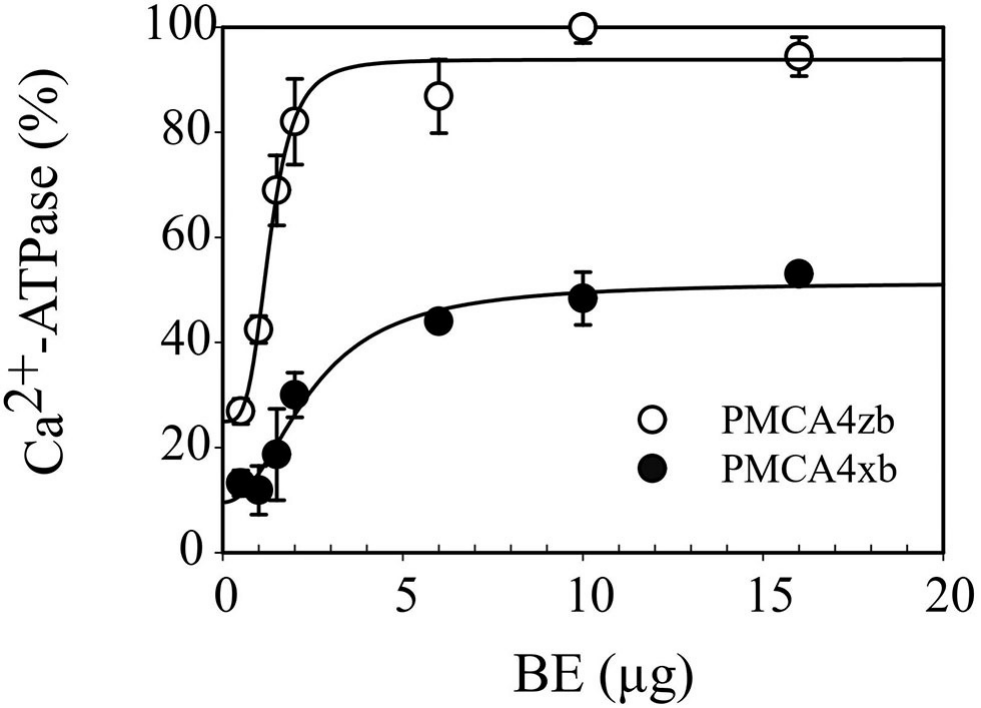
Activation of purified PMCA4xb-GFP and PMCA4zb-GFP by acidic lipids. The Ca^2+^-ATPase activity was measured as indicated in Figure 5. PMCA4xb-GFP and PMCA4zb-GFP proteins (1 μg) were supplemented with the indicated amount of acidic lipid brain extract (BE). The concentration of Ca^2+^ was 10 μM. The continuous lines represent the best fitting of the data to a Hill equation *v* = *v*_0_ + *v*_BE_ *[BE]^nH^ /([K_0.5BE_]^nH^ + [BE]^nH^) with the following parameters. For PMCA4xb, *v*_0_ = 9 ± 5%, *v*_BE_ = 42 ± 8%, K_0.5BE_ = 2.4 ± 0.5 μg, nH = 2 ± 1; for PMCA4zb; *v*_0_ = 25 ± 7%, *v*_BE_ = 69 ± 7%, K_0.5BE_ = 1.3 ± 0.1 μg, nH = 4 ± 1.

To compare the responses of PMCA4xb and PMCAzb to acidic lipids, we supplemented the purified proteins with different amounts of a mixture of acidic lipids extracted from the porcine brain (BE) before measuring the Ca^2+^-ATPase activity. As shown in Figure 6, the activity of both PMCA4xb and PMCAzb increased with increasing amounts of BE following a sigmoidal curve. At saturating, amounts of BE PMCA4zb had about two times the activity of PMCAxb, an increment somewhat similar to that obtained with calmodulin. However, at lower amounts of acidic lipids, PMCA4zb was nearly 8-fold more active than PMCA4xb, and its activity increased steeply, reaching maximal activity at lower amounts of BE.

## Discussion

The existence of a great diversity of PMCA isoforms and splice variants is likely the result of cell-specific requirements for controlling the spatial and temporal magnitude of the cytosolic Ca^2+^ signaling. Most cells express simultaneously a variety of PMCA variants, and for understanding the role of PMCA, it is necessary to advance the knowledge of each PMCA protein, their basal activity and how they respond to activators (Brini et al., 2003; Streheler et al., 2007). PMCA4 is a ubiquitously expressed isoform. Previous studies of PMCA4 used the protein from natural sources, for example, red blood cells which in addition contain PMCA1, or with the recombinant PMCA4x protein (Enyedi et al., 1994; Adamo and Grimaldi, 1998; de Tezanos Pinto and Adamo, 2002, 2006; Bredeston and Adamo, 2004; Corradi and Adamo, 2007; Cura et al., 2008; Mazzitelli and Adamo, 2014). In this study, we have focused on the two PMCA4 variants “x” and “z” produced by the splice at site A, we compared the kinetics properties of purified micellar preparations of the PMCA4xb and PMCA4zb obtained by expression in yeast cells.

Recent studies showed that PMCA associates with IgG-domain proteins neuroplastin and basigin (Korthalsa et al., 2017; Schmidt et al., 2017). The interaction with these auxiliary subunits influences PMCA function, stability, and membrane targeting. The PMCA-neuroplastin interaction seems quite sensitive to the detergents used for PMCA purification, a fact that may explain why the neuroplastin peptide was not previously identified in purified preparations of PMCA despite the proposed equimolar association PMCA-neuroplastin (Schmidt et al., 2017). In this work, as in previous studies of recombinant PMCA, we expressed only the catalytic PMCA4 polypeptide.

### PMCA Expression in Yeast

As we reported previously, yeasts are capable of functional PMCA expression (Bredeston and Adamo, 2004; Cura et al., 2008; Mazzitelli and Adamo, 2014). Active PMCA can complement the K616 yeast, and the purified recombinant protein is fully functional. This seems somewhat puzzling because yeasts lack an obvious neuroplastin homolog. However, we observed that expressed PMCA4xb and PMCA4zb localized in the yeast ER membrane, a fact that might be related to the lack of yeast neuroplastin. Further studies of the importance of neuroplastin for PMCA targeting, function, and regulation seem necessary.

The expression of the wild-type autoinhibited PMCA4xb does not rescue the yeast K616 phenotype (Bredeston and Adamo, 2004; Mazzitelli and Adamo, 2014). Successful K616 complementation requires the activation of PMCA4xb by mutations that disrupt the autoinhibitory mechanism. On the contrary, we found that K616 cells expressing the variant PMCA4zb did proliferate in Ca^2+^ depleted media. Thus, according to the K616 complementation assay, PMCA4zb has a higher Ca^2+^ transporting activity than PMCA4xb in the yeast cell. In addition, we determined that the purified PMCA4zb had higher Ca^2+^-ATPase activity and higher apparent affinity for Ca^2+^ than PMCA4xb in the absence of any activator, confirming the idea that PMCA4zb is a naturally hyperactive PMCA4 variant. Calmodulin activated both PMCA4 variants and increased their affinity for Ca^2+^ to a high and similar value. This result suggests that the different Ca^2+^ affinity of the two variants in the absence of calmodulin should be related to the mechanism of autoinhibition and does not involve the modification of the intrinsic affinity of the Ca^2+^ binding site.

### Acidic–Lipid stimulation

Yeast expression provides preparations of purified PMCA, which are very sensitive to acidic lipids (Cura et al., 2008). We found that acidic lipids stimulated both yeast recombinant PMCA4 variants but the activity of PMCA4zb increased much more rapidly with low amounts of acidic lipids. The activation by acidic lipids would involve the interaction at two distinct sites of the PMCA molecule, one in the calmodulin-binding site at the C-terminal regulatory segment, and the second in the A_L_ region of the A-M3 linker (Enyedi et al., 1994; de Tezanos Pinto and Adamo, 2002, 2006). In PMCA4xb, the linker A-M3 region comprises the segment of amino acid residues 300–356. A peptide from this region (residues 339–363) binds acidic lipids, although with a lower affinity than the calmodulin-binding site (Brodin et al., 1992; Filoteo et al., 1992). Thus, the primary site of acidic lipid activation would overlap with that of calmodulin.

We have previously characterized mutants of PMCA4xb containing deletions in the A_L_ region using microsomes of CHO cells expressing the recombinant proteins (de Tezanos Pinto and Adamo, 2002, 2006). The deletion mutant d300-314, with a sequence similar to the natural splicing A variant PMCA4zb, exhibited a higher Ca^2+^ transport activity due to the higher apparent affinity for Ca^2+^. On the contrary, deletion of the downstream region (residues 339–360), which was indicated as the acidic lipid-binding site (Brodin et al., 1992), did not promote the activation of the PMCA. While the results presented here show that splice A alters the acidic–lipid stimulation of PMCA4, there is no conclusive evidence that this effect was mediated by the direct binding of lipids to residues 339–360.

The atomic model of PMCA1d was recently reported, but unfortunately, the structure of the A-M3 linker could not be determined (Gong et al., 2018). This segment is enriched in polar and charged residues, and as judged by its susceptibility to protease cleavage, is unusually accessible (Brodin et al., 1992). In this line, it is worth considering that PMCA is very sensitive to cellular proteases and most PMCA preparations exhibit some proteolysis (Adamo et al., 2000), a fact that has led to propose that proteolysis is a natural mechanism for regulating PMCA (Filoteo et al., 1997). We detected a small number of proteolytic fragments in both the PMCA4xb and the PMCAzb preparations. By using the GFP fusions and antibodies with defined epitopes, we observed that the endogenous proteolytic pattern of the PMCA4xb and the PMCA4zb was similar. In addition, the main proteolytic fragment corresponded to the full-length proteins lacking GFP suggesting that a cleavage near the C-terminus of the protein occurred without affecting the C-terminal regulatory region.

It seems appropriate to notice that the variability produced by the splicing site A may have different consequences depending on the PMCA isoform considered. Indeed, in polarized cells, the splice variant “w” targets PMCA2 to apical membranes, while variants “x” and “z” remain basolateral (Chicka and Strehler, 2003; Antalffy et al., 2012). On the other hand, PMCA4x is targeted to apical membranes in pancreatic acinar cells indicating that the targeting capacity of splice A variants may be isoform and cell-specific (Baggaley et al., 2007). Furthermore, membranes from CHO cells expressing PMCA2wb and PMCA2xb exhibit similar Ca^2+^-ATPase activity and acidic lipid stimulation suggesting that the “w” insertion in PMCA2 had minimal functional consequences (Brini et al., 2010). Interestingly, the PMCA2zb was recognized as a very active pump capable of decreasing the Ca^2+^ signal peak in about half the time needed by the PMCA2wb (Ficarella et al., 2007).

### A Possible Molecular Mechanism of PMCA4zb Activation

The A-M3 linker where the splicing A variants of PMCA occur is an important functional region of the P-ATPases. Indeed, the rotation of the A domain that accompanies the formation of an intramembrane exit pathway of the bound transported substrate is triggered by the strain of the A-M3 (Nagarajan et al., 2012). The functional consequences of the A-M3 linker length were analyzed in detail in SERCA (Nyholm Holdensen and Andersen, 2009). SERCA possesses a shorter A-M3 linker compared with the PMCA (refer to Figure 1). A series of insertional SERCA mutants containing up to 41 glycine and proline residues at the corresponding site of splicing A of PMCA, decreased the SERCA turnover to 30% of the WT. These results show that, in analogy with SERCA, the length of the A-M3 linker influenced the activity of the PMCA and made PMCAzb with a shorter linker more active than PMCA4x.

**Figure 7:**
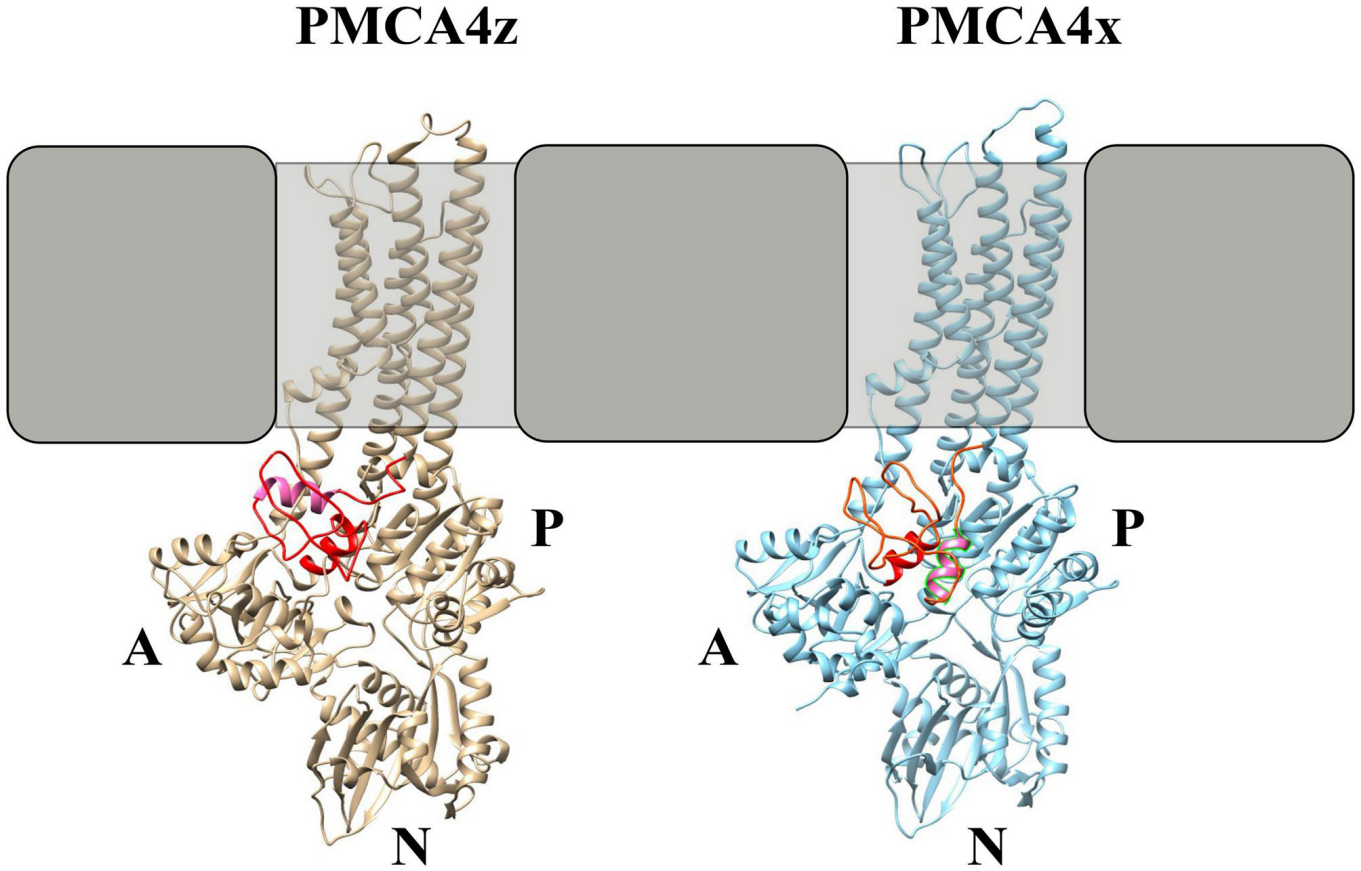
Molecular modeling of PMCA4x and PMCA4z. Homology models of PMCA4 were obtained using the partial structure of PMCA1d (Gong et al., 2018) as a template. Only the portion of the PMCA from the amino-terminal region to the end of M6 was modeled. The tertiary structure and contact prediction model of the A-M3 linker were obtained using the RaptorX web service (Källberg et al., 2012). The SERCA-conserved A3 helix is colored red while the putative A_L_ helix in PMCA is colored pink.

In comparison with SERCA, the PMCA A-M3 linker shows, in addition to the conserved A3 helix, a possible helical structure highly enriched in charged amino acids formed by PMCA4x residues E335-V342. By using a combination of homology modeling and structure prediction (Källberg et al., 2012), it can be observed that in PMCA4x these helical structures are deeply inserted in the catalytic core, where they may impair the interaction of the A and P domains while in PMCA4z these helices would move toward the surface of the protein (Figure 7).

A clear difference between SERCA and PMCA pumps is the C-terminal autoinhibitory domain of PMCA that binds to the A and N domains. A lower affinity of the autoinhibitory domain for its sites in the core of the protein may explain the basal activated state of PMCA4zb. It seems reasonable to consider that two activating mechanisms, one involving the modification of the A-M3 linker (splice site A) and the other resulting in the release of the C-terminal autoinhibition (splice site C) are not independent but cooperate for regulating the activity of the PMCA.

In this study, we found that PMCA4z a previously not characterized variant of PMCA originated by splicing at site A is more active and more sensitive to activation by acidic lipids than the more common form of PMCA4x. Future work will be needed to assess the relevance of this finding for the control of the Ca^2+^ signal in cells expressing the variant “z” in normal and pathological conditions.

## Data Availability Statement

The raw data supporting the conclusions of this article will be made available by the authors, without undue reservation.

## Author Contributions

GRC, LRM, GDP, LR, and FTP performed experiments and analyzed data. GRC, FTP, LRM, and HPA designed research and analyzed data. HPA conceived the project and wrote the manuscript with comments from all the authors. All authors contributed to the article and approved the submitted version.

## Conflict of Interest

The authors declare that the research was conducted in the absence of any commercial or financial relationships that could be construed as a potential conflict of interest.

## Publisher's Note

All claims expressed in this article are solely those of the authors and do not necessarily represent those of their affiliated organizations, or those of the publisher, the editors and the reviewers. Any product that may be evaluated in this article, or claim that may be made by its manufacturer, is not guaranteed or endorsed by the publisher.

## Abbreviations

PMCA: plasma membrane Ca^2+^ pump
PMCA4xb: plasma membrane Ca^2+^ pump derived from the human gene 4, spliced form ***x*** at the splicing site “A” and spliced form ***b*** at the splicing site “C”
PMCA4zb: plasma membrane Ca^2+^ pump derived from the human gene 4, spliced form z at the splicing site “A” and spliced form ***b*** at the splicing site “C”
GFP: green fluorescent protein, CaM, calmodulin
PC: phosphatidylcholine
PI: phosphatidylinositol
PS: phosphatidylserine
PA: phosphatidic acid
C_12_E_10_: polyoxyethylene10-laurylether
E_1_ and E_2_: conformers of the Ca^2+^-ATPase
EP: phosphoenzyme
SDS-PAGE: sodium dodecyl sulfate-polyacrylamide gel electrophoresis
BE: extract from bovine brain containing acidic lipids.
